# Multi-Architecture Deep Learning for Early Alzheimer’s Detection in MRI: Slice- and Scan-Level Analysis

**DOI:** 10.3390/ijerph23030322

**Published:** 2026-03-05

**Authors:** Isabelle Bricaud, Giovanni Luca Masala

**Affiliations:** School of Computing, University of Kent, Canterbury CT2 7PE, UK

**Keywords:** Alzheimer’s disease, medical imaging, structural MRI, deep learning, convolutional neural networks, vision transformers, transfer learning, multi-slice evaluation, ADNI dataset, early Alzheimer’s detection

## Abstract

**Highlights:**

**Public health relevance—How does this work relate to a public health issue?**

- Alzheimer’s disease is a major global public health challenge, causing substantial mortality, disability, and economic and caregiving burden in ageing populations.
- Alzheimer’s disease is the most common cause of dementia and is associated with progressive memory and cognitive decline.

**Public health significance—Why is this work of significance to public health?**

- Earlier and more reliable detection may slow disease progression, preserve independence, and reduce long-term healthcare and societal costs.
- Emphasising standardised MRI image preprocessing improves the reliability and reproducibility of automated diagnostic tools for population-level use.

**Public health implications—What are the key implications or messages for practitioners, policy makers and/or researchers in public health?**

- AI-assisted MRI analysis may assist clinicians in identifying high-risk individuals earlier, particularly where specialist resources are limited.
- Investment in standardised neuroimaging pipelines can support scalable, equitable early detection strategies for Alzheimer’s disease.

**Abstract:**

Alzheimer’s disease (AD), the most common form of dementia, is a progressive and irreversible neurodegenerative disorder. Structural MRI is widely used for diagnosis, revealing brain changes associated with AD. However, these alterations are often subtle and difficult to detect manually, particularly at early stages. Early intervention during prodromal stages, such as mild cognitive impairment (MCI), can help slow disease progression, highlighting the need for reliable automated methods. In this work, we introduce a dual-level evaluation framework comparing fifteen deep learning architectures, including convolutional neural networks (CNNs), Transformers, and hybrid models, for classifying AD, MCI, and cognitively normal (CN) subjects using the ADNI dataset. A central focus of our work is the impact of robust and standardized preprocessing pipelines, which we identified as a critical yet underexplored factor influencing model reliability. By evaluating performance at both slice-level and scan-level, we reveal that multi-slice aggregation affects architectures asymmetrically. By systematically optimizing preprocessing steps to reduce data variability and enhance feature consistency, we established preprocessing quality as an essential determinant of deep learning performance in neuroimaging. Experimental results show that CNNs and hybrid pre-trained models outperform Transformer-based models in both slice-level and scan-level classification. ConvNeXtV2-L achieved the best scan-level performance (91.07%), EfficientNetV2-L the highest slice-level accuracy (86.84%), and VGG19 balanced results (86.07%/88.52%). ConvNeXtV2-L and SwinV1-L exhibited scan-level improvements of 7.60% and 9.04% respectively, while EfficientNetV2-L experienced degradation of 2.66%, demonstrating that architectural selection and aggregation strategy are interdependent factors. These findings suggest that carefully designed preprocessing not only improves classification accuracy but may also serve as a foundation for more reproducible and interpretable Alzheimer’s disease detection pipelines.

## 1. Introduction

The most common cause of dementia is Alzheimer’s disease (AD), which results in the loss of memory, mental, and intellectual abilities. It is a progressive neurodegenerative disorder characterized by the degeneration of healthy brain cells, the formation of plaques and brain tissue atrophy. This disease mostly affects the elderly population over age 65, with only 3% of dementia cases occurring in younger individuals [1]. Dementia is currently the seventh leading cause of death and one of the major causes of disability and dependency among older people [2]. It ranks as the fourth leading cause of death in developed countries [3]. In 2021, an estimated 57 million people worldwide were affected by dementia, including AD, with nearly 10 million new cases occurring annually [2]. This number may more than double by 2050, reaching approximately 139 million dementia patients [1]. In 2019, dementia cost the world economy US$1.3 trillion. Approximately half of these costs reflect unpaid care provided by family members and close friends, who spend an average of 5 h daily caring for and supervising individuals with dementia [2]. Dementia disproportionately affects women both directly and indirectly. Women experience higher disability-adjusted life years and mortality due to dementia. Additionally, they assume the majority of caregiving responsibilities, contributing approximately 70% of total care hours [2]. While Alzheimer’s disease deeply affects those who live with it, its impact reaches far beyond the individual. The illness places heavy economic and emotional demands on patients, families and healthcare systems. More than 11 million family members and other unpaid caregivers provided an estimated 18 billion hours of care to people with Alzheimer’s or other dementias in 2022, valued at $339.5 billion [4]. These figures reflect both a decline in caregiver numbers compared to a decade earlier and an increase in care hours per remaining caregiver [4]. Earlier detection can ease these challenges by maintaining cognitive health, prolonging independence and reducing the need for long-term care. The actual rate of missed or delayed diagnoses remains unclear but is likely substantial, with contributing factors including communication gaps, educational deficits, and limited healthcare resources [5].

From a public health perspective, early detection stands out as the most effective means of slowing the progression of the disease across society. A critical focus in AD research is on Mild Cognitive Impairment (MCI), an intermediate stage between normal cognition and AD, where subtle brain changes occur without clear symptoms. The biological changes that lead to Alzheimer’s disease are believed to start many years before symptoms appear, providing a crucial opportunity for therapeutic intervention during this long preclinical phase [6]. Studies show that 10–15% of MCI patients progress to AD annually [7]. With no current cure for AD and medications only slowing progression, early detection is fundamental to reducing the number of affected individuals [7,8].

Early intervention when memory problems first appear, particularly during mild cognitive impairment (MCI), is crucial for slowing neurodegenerative disease progression. Research demonstrates that treatments are most effective when initiated early, before extensive brain damage occurs. Because Alzheimer’s disease develops slowly over many years before symptoms appear, this early stage offers a promising opportunity for intervention, when new treatments may have their greatest effect [6]. As of 2020, 121 agents were in clinical trials for AD treatment, including 97 in disease modification trials. There is progressive emphasis on interventions addressing neuroinflammation, synaptic and neuronal protection, and vascular contributions to the disease [9]. As the disease progresses and brain cells deteriorate, treatments become less effective, making it increasingly difficult to slow cognitive decline. Missed or delayed diagnoses forfeit valuable intervention opportunities when treatment could have the greatest impact. Early intervention provides patients the best opportunity to preserve cognitive abilities and maintain independence in daily life.

Neuroimaging, particularly Magnetic Resonance Imaging (MRI) is a non-invasive and effective tool that stands out to detect the early disease by visualizing structural changes within the tissues of the brain [1,3]. Special attention is given to the hippocampal region on axial MRI slices, which provides detailed visualization of areas affected by AD-related atrophy [10,11]. Brain atrophy rates measured from serial MRI studies are associated with cognitive decline in both healthy elderly subjects and those with amnestic MCI [12]. Higher whole-brain and ventricular atrophy rates are associated with increased risk of MCI-to-AD conversion. Combining baseline hippocampal volume measures with atrophy rates from serial MRI scans provides complementary predictive information [12]. These changes may be imperceptible to the human eye, which is why studies increasingly employ deep learning techniques to address limitations of manual diagnosis. Deep learning, particularly CNNs have shown excellent results in analysing complex MRI images and achieving high classification accuracy [13,14].

Structural alterations associated with AD are often subtle and difficult to detect manually, especially at early stages, highlighting the need for reliable automated methods. While deep learning approaches show promise, preprocessing quality remains a critical yet underexplored factor influencing model reliability. This work implements and compares fifteen deep learning architectures for classifying cognitively normal (CN), MCI, and AD subjects using the ADNI dataset. A central focus is the preprocessing pipeline including skull stripping, bias field correction, tissue segmentation, spatial normalization, and slice extraction to reduce data variability. We demonstrate that preprocessing quality is a key determinant of deep learning performance in neuroimaging, providing a foundation for more reproducible and interpretable AD detection pipelines.

While deep learning approaches show promise for AD classification, the interaction between architectural design and evaluation granularity remains critically underexplored. Most studies report either slice-level or scan-level performance without systematically investigating how aggregation affects different architectures. Additionally, heterogeneous preprocessing and inconsistent data splitting strategies complicate cross-study architectural comparisons, obscuring whether performance differences stem from architectural design or methodological variations.

This work addresses these gaps through a dual-level evaluation framework that quantifies both slice-level and scan-level performance across fifteen architectures under rigorously controlled conditions with scan-based splitting and standardized preprocessing. Our methodological contribution lies in revealing architecture-dependent aggregation behavior and providing a transparent baseline for reproducible comparison.

This article is organized as follows: Section 2 details the dataset and cohort study, describes the preprocessing pipeline and presents the deep learning models evaluated. Section 3 reports results at slice- and scan-level and comparison with literature. Section 4 discusses health promotion impact and study limitations. Finally, Section 5 concludes with synthesis of key findings and future work directions for early AD detection.

## 2. Materials and Methods

This section describes the dataset and the cohort study, the preprocessing pipeline, the evaluated architectures and the performance evaluation metrics.

### 2.1. Dataset and Cohort Study

All neuroimaging data were obtained from the Alzheimer’s Disease Neuroimaging Initiative (ADNI) [15] database, a public-private partnership established in 2004 encompassing five phases (ADNI1, ADNI-GO, ADNI2, ADNI3, and ADNI4). Data access requires approval. We extracted structural MRI scans from the ADNI1 cohort spanning three years of longitudinal follow-up. The study population comprised 639 participants aged 55–93 years (mean age 76.23 ± 6.72 years), including 195 cognitively normal (CN) individuals, 311 patients with mild cognitive impairment (MCI), and 133 patients diagnosed with Alzheimer’s disease (AD). The gender distribution consisted of 267 women and 372 men (Table 1). Many participants underwent multiple scanning sessions at baseline, 6 months, 1 year, 18 months, 2 years, and 3 years. Each scan received diagnostic classification (CN, MCI, or AD) based on clinical evaluation at the time of acquisition. A total of 3134 MRI scans were collected: 984 CN, 1575 MCI, and 575 AD.

All brain MRI scans were acquired using 1.5 Tesla scanners from different manufacturers including General Electric Healthcare, Philips Medical Systems and Siemens Medical Solutions, following the standardized ADNI MRI acquisition protocol. Images were acquired using the Magnetization-Prepared Rapid Gradient-Echo (MPRAGE) protocol, a T1-weighted sequence optimized for high-resolution anatomical imaging. All data were provided in Neuroimaging Informatics Technology Initiative (NIfTI) format as complete 3D volumes. Acquisition parameters varied across scanners but typically included: field strength 1.5 T, flip angle 8°, slice thickness ∼1.2 mm, sagittal acquisition plane, voxel dimensions 1.25×1.25×1.2 mm or 1.0×1.0×1.2 mm, matrix dimensions 192×192 or 256×256 pixels, and 160–256 slices along the Z-axis depending on head size and positioning.

### 2.2. Preprocessing Pipeline

**Figure 1:**
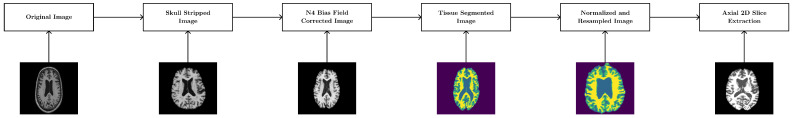
Preprocessing pipeline flowchart showing sequential preprocessing steps.

We implemented a preprocessing pipeline to ensure data quality, consistency, and harmonization across all MRI volumes, preparing the dataset for model training and evaluation. The complete preprocessing pipeline integrating all stages is summarized in Figure 1 illustrating all transformations applied from raw ADNI MRI image to the 2D slice dataset.

Each preprocessing step addresses specific sources of variability that would otherwise confound feature learning. Skull stripping removes extracranial tissues, which exhibit scanner- and protocol-dependent intensity patterns but provide no diagnostic information for neurodegenerative disease. Bias field correction addresses spatial intensity inhomogeneities caused by magnetic field imperfections, which make identical tissue types appear with different intensities at different locations. Tissue segmentation provides explicit anatomical context by separating cerebrospinal fluid, gray matter, and white matter, allowing models to learn tissue-specific atrophy patterns. Spatial normalization to MNI space removes subject-specific anatomical variability in brain size, shape, and positioning, ensuring identical voxel coordinates represent homologous anatomical locations across subjects. Anatomically guided slice extraction focuses analysis on hippocampal regions exhibiting early AD-related atrophy while preserving sufficient anatomical context. While we followed established best practices, we did not conduct ablation studies to quantify each step’s contribution to final performance, a limitation we acknowledge for future investigation.

#### 2.2.1. Skull Stripping

**Figure 2:**
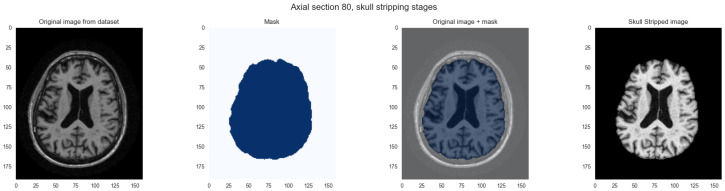
Skull stripping process on axial section. From left to right: (1) Original MRI image. (2) Mask generated by DeepBrain library. (3) Original image with mask overlay delineating brain tissue boundaries. (4) Final skull stripped image containing only cerebral tissues.

Skull stripping removes non-brain tissues considered noise that can degrade downstream processing performance. By isolating brain tissue, skull stripping ensures subsequent analysis focuses exclusively on relevant structures. We performed this step using the DeepBrain library (https://github.com/iitzco/deepbrain, accessed on 16 June 2025). This tool employs a pre-trained neural network that analyzes each voxel in a 3D MRI volume to predict whether it represents brain tissue or extracranial structures. The DeepBrain network uses a U-Net encoder-decoder architecture trained on expert-annotated masks, applying probability thresholding at 0.5 and morphological post-processing to ensure mask connectivity. These probabilities are converted to binary masks distinguishing brain from non-brain tissue. Applying these masks to the original MRI volumes yields skull-stripped images preserving only brain tissue. Figure 2 illustrates the skull stripping process applied to MRI images. This deep learning approach adapts more robustly to anatomical variations than intensity-based methods and reduces scanner-specific variance that might otherwise dominate learned features in shallow network layers.

#### 2.2.2. N4 Bias Field Correction

**Figure 3:**
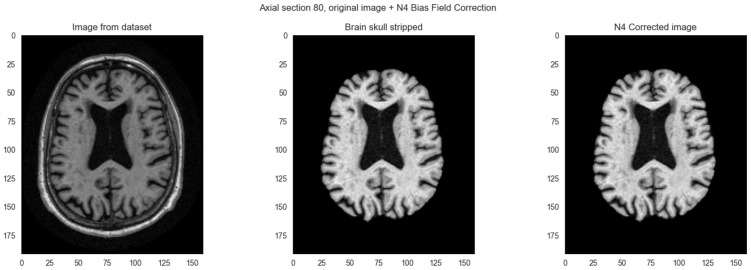
N4 bias field correction on axial section. From left to right: (1) Original MRI image. (2) Skull stripped image showing residual non-uniformity in intensity. (3) Bias field corrected image with normalized intensity across brain regions.

Bias field artifacts are common in medical imaging, manifesting as undesirable intensity variations across images. Consequently, identical tissue types may appear lighter or darker depending on spatial location due to magnetic field imperfections during acquisition. We corrected these intensity variations using the SimpleITK library’s N4ITK (N4 Insight Toolkit) [16] algorithm. N4ITK models the bias field as a smooth B-spline function and estimates it iteratively in the logarithmic domain using a multi-resolution approach [17,18]. To reduce computational cost, the algorithm first estimates the bias field on a downsampled image (shrink factor 2–4) before reconstructing it at full resolution. Final correction applies voxel-wise division of the original image by the estimated bias field, standardizing tissue intensities across spatial locations. Figure 3 illustrates the effect of N4 bias field correction. Without this correction, models might exploit position-dependent intensity gradients from field inhomogeneity rather than learning anatomically meaningful tissue patterns.

#### 2.2.3. Tissue Segmentation

**Figure 4:**
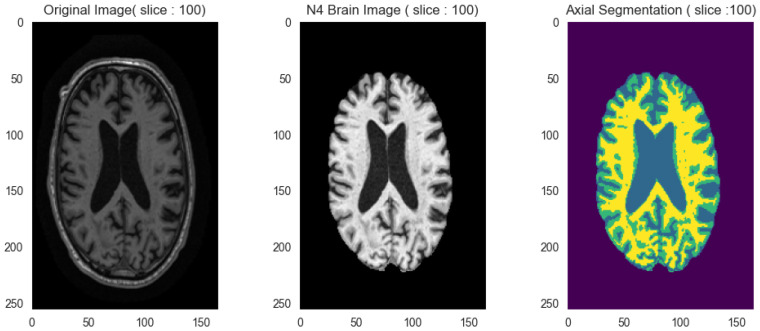
Brain tissue segmentation on axial section. From left to right: (1) Original MRI image. (2) Bias field corrected image. (3) Tissue segmentation result where CSF is depicted in blue, GM in green and WM in yellow.

We performed tissue segmentation using the Hidden Markov Random Field (HMRF) algorithm implemented in the DIPY library [19,20]. Using a Bayesian approach with T1-weighted MRI images, the method classifies each brain voxel into cerebrospinal fluid (CSF), white matter (WM), or gray matter (GM). The observation model defines the likelihood term using Gaussian distributions characterizing each tissue type’s intensity values. Optimization uses Expectation-Maximization (EM) coupled with Iterated Conditional Modes (ICM), typically converging within 10–20 iterations. A Markov Random Field (MRF) prior encourages spatial smoothness through neighbor regularization. In our pipeline, tissue segmentation serves primarily as an intermediate step that enhances spatial normalization by providing clear anatomical landmarks for registration. Figure 4 illustrates the resulting tissue probability maps.

#### 2.2.4. Spatial Normalization and Resampling

**Figure 5:**
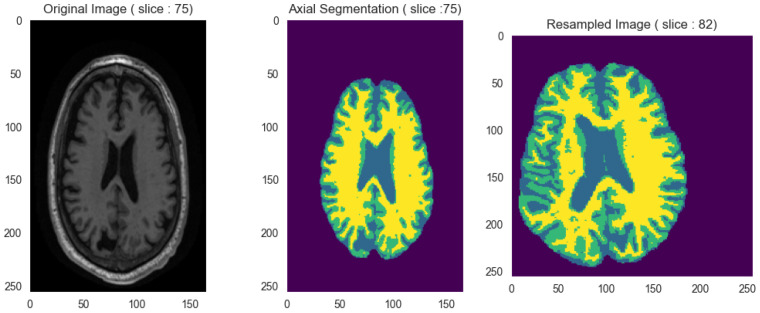
Spatial normalization and resampling process on axial section. From left to right: (1) Original MRI image with variable dimensions. (2) Tissue segmented image. (3) Registered and resampled image in MNI space.

Substantial anatomical variability across subjects and acquisition protocols necessitated spatial normalization to a standard reference space. We used the Montreal Neurological Institute (MNI) 152 template (MNI152_T1_1mm.nii) [21,22] as the target reference atlas. This template provides a population-representative anatomical framework that serves as a universal coordinate system in neuroimaging. Registration employed a centered 3D affine transformation for geometric alignment between the segmented brain MRI and the MNI template. Image alignment used Mattes Mutual Information [23], a metric quantifying shared information between the moving and fixed images. Optimization employed gradient descent combined with a pyramidal multi-resolution approach. Different shrink factors and progressively decreasing Gaussian smoothing levels guided the algorithm toward the global optimum while preventing premature convergence. After registration, we resampled all volumes to uniform 1 mm^3^ isotropic resolution with standardized 256×256×256 matrix dimensions using nearest-neighbor interpolation. This step ensured consistent spatial normalization across the dataset while preserving scan integrity. Figure 5 demonstrates the registration and resampling process.

#### 2.2.5. Axial 2D Slice Extraction

Prior literature identifies the hippocampus, entorhinal cortex, parahippocampal cortices, and lateral ventricles as key AD biomarker regions. Following Lim et al. [8], who reported that slices within the 160–170 index range provided optimal representation of gray matter (GM), white matter (WM), and cerebrospinal fluid (CSF) patterns for AD classification, we adapted this approach to MNI-normalized space. Because MNI normalization alters slice indices, we used anatomical landmark alignment to identify the corresponding axial level in template space. The z = 60 mm coordinate in MNI space consistently intersects the medial temporal lobe across subjects, including hippocampal formation and adjacent ventricular structures. This axial plane enables simultaneous visualization of bilateral hippocampi, lateral ventricle enlargement, and well-defined GM/WM/CSF boundaries after preprocessing. We selected the axial orientation [24] because it captures bilateral medial temporal structures within a single plane, advantageous for 2D CNN-based analysis.

To address class imbalance, generate sufficient training data, and capture regional anatomical variability, we extracted variable numbers of slices per scan: 11 for CN (10,824 images), 7 for MCI (11,025 images), and 18 for AD (10,350 images), totaling 32,199 grayscale images (Table 2). This extraction strategy reduced sensitivity to minor registration misalignments or individual anatomical variability by representing hippocampal and surrounding temporal structures across multiple adjacent planes. Our preprocessing pipeline ensured clear GM, WM, and CSF boundary representation, enabling models to learn biologically meaningful patterns including hippocampal atrophy, cortical thinning, and ventricular enlargement. We designed the variable extraction strategy to balance the training dataset given unequal scan distribution (984 CN, 1575 MCI, 575 AD).

**Figure 6:**
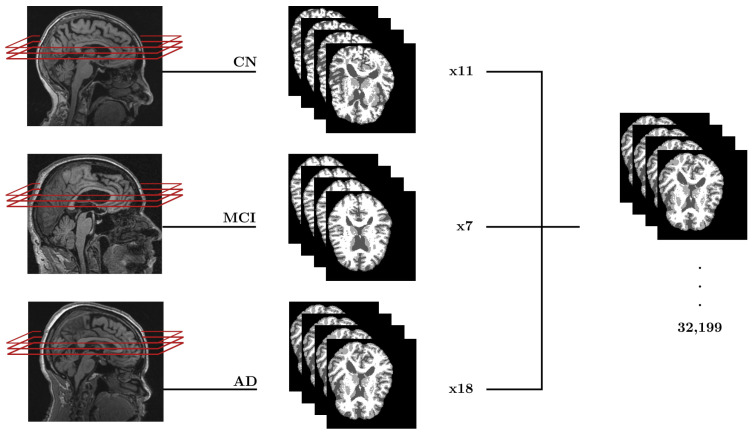
Axial slice extraction pipeline from 3D MRI images to 2D dataset. Sagittal views show slice extraction with symmetric offsets. Each diagnostic category (CN, MCI, AD) yields multiple axial slices per scan, totalling 32,199 grayscale images constituting the final classification dataset.

All slice extractions followed systematic symmetric patterns around the central z = 60 mm position. For odd slice counts, we included the central slice plus symmetric pairs at 2 mm intervals (e.g., z = 60, 58/62, 56/64 mm). For even counts, we excluded the central position and extracted only symmetric pairs. This ensured consistent spatial sampling relative to the hippocampus and other anatomical regions across classes. We normalized each extracted slice to the 0–255 range for PNG format, reducing storage requirements from 215 GB (original NIfTI volumes) to 116 MB (extracted PNG slices). Figure 6 illustrated the complete slice extraction pipeline.

### 2.3. Data Splitting Strategy

To ensure proper evaluation and limit data leakage, we implemented scan-based splitting rather than image-based randomization. This approach ensured that all slices from the same MRI scan belonged exclusively to a single set, preventing artificially inflated performance from high inter-slice correlations.

The ADNI cohort contains multiple longitudinal scans per subject acquired at different timepoints (baseline, 6, 12, 18, 24, and 36 months). Our scan-level splitting strategy did not enforce subject-level separation, meaning different visits from the same individual could appear across sets, introducing potential temporal overlap.

We conducted data splitting in two stages. First, we extracted scan identifiers from filenames and grouped all slices from the same scan. We then randomly shuffled these scan identifiers and divided them using an 80:20 ratio to create training and test sets, ensuring complete scan-level separation. Second, we subdivided the training set into training and validation subsets using another 80:20 split. To address memory constraints, we used the TensorFlow Data API (tf.data, v2.16.1) for batch loading rather than loading all images simultaneously into RAM.

Preliminary k-fold cross-validation experiments (k = 3, 5, 10) on selected architectures revealed low performance variance across folds (standard deviation ∼ 0.03), indicating our test set size provided stable estimates. Given this minimal variability and the computational cost of k-fold validation across fifteen architectures, we employed single train-validation-test splitting with early stopping, ensuring robust evaluation while enabling comprehensive architectural comparison.

### 2.4. Deep Learning Architectures

We implemented and evaluated fifteen deep learning architectures to identify optimal approaches for three-way classification of cognitively normal (CN), mild cognitive impairment (MCI), and Alzheimer’s disease (AD) subjects. Our architecture selection encompasses three major categories: (i) traditional convolutional neural networks (CNNs), (ii) Vision Transformer models employing self-attention mechanisms, and (iii) hybrid models representing modern convolutional architectures incorporating transformer-inspired design principles. We evaluated the following fifteen models, whose characteristics and implementation details are summarized in Table 3.

All architectures except the custom CNN utilized transfer learning, leveraging pre-trained weights from large-scale image datasets to improve medical imaging performance. We adapted each model for three-way classification through custom classification heads outputting probability distributions across diagnostic categories. We maintained pre-trained feature extraction backbones to preserve learned representations while adapting to medical imaging characteristics.

### 2.5. Top-Performing Architectures

Three models achieved the highest performance while demonstrating optimal balance between diagnostic accuracy and implementation feasibility. We provide detailed descriptions of these three models below. We trained all models using early stopping callbacks that monitored validation loss with patience periods to prevent overfitting and ensure convergence. Rather than imposing uniform epoch counts, we allowed each model to train until performance plateaued, ensuring fair comparison based on optimal achievable accuracy rather than arbitrary training duration.

#### 2.5.1. VGG19 Architecture

VGG19 is a deep convolutional neural network architecture consisting of sixteen convolutional layers organized in five blocks with progressively increasing filter counts of 64, 128, 256, 512, and 512 filters respectively, followed by three fully connected layers [25].

We used a pre-trained VGG19 model [25,26] to extract features from MRI images. We replaced the model’s final layers with a custom classification head for three-way classification (AD, MCI, CN).

#### 2.5.2. EfficientNetV2-L Architecture

EfficientNetV2-L represents a convolutional neural network architecture utilizing Fused-MBConv blocks in early stages and MBConv blocks incorporating Squeeze-and-Excitation mechanisms in later stages, structured across seven successive stages [27].

We used a pre-trained EfficientNetV2-L model [27,28] to extract features from MRI images. We replaced the original classification layers with a custom head for three-way diagnostic classification.

#### 2.5.3. ConvNeXtV2-L Architecture

ConvNeXtV2-L is a hybrid architecture combining traditional convolutional neural networks and incorporating the design principles of Vision Transformers. The architecture consists of an initial stem block, followed by four stages containing ConvNeXtV2 blocks [29].

We used a pre-trained ConvNeXtV2-L model [29] to extract features from MRI images. We adapted the model’s final output layer for three-way diagnostic classification.

### 2.6. Evaluation Metrics and Methodology

We evaluated model performance using multiple metrics computed on the independent test set for comprehensive assessment of three-way classification performance. We computed standard classification metrics (accuracy, precision, recall, and F1-score [30]) for each class. Accuracy represents the proportion of correct predictions across all classes. Precision reflects the proportion of correct predictions among all samples assigned to a specific class. Recall (sensitivity) quantifies the proportion of true class instances correctly detected, indicating the model’s ability to identify patients in a particular diagnostic category while minimizing missed diagnoses. The F1-score, computed as the harmonic mean of precision and recall, balances the cost of false positives and false negatives. We used categorical cross-entropy loss to quantify divergence between predicted probability distributions and true class labels.

We implemented two evaluation strategies to assess model performance from complementary clinical perspectives. In slice-level classification, we treated each extracted 2D axial slice as an independent sample. For a given slice *s*, the model computed a probability distribution $P\left(s\right)=\left[p_{\mathrm{CN}},p_{\mathrm{MCI}},p_{\mathrm{AD}}\right]$, where $p_{\mathrm{CN}},p_{\mathrm{MCI}}$ and $p_{\mathrm{AD}}$ denote the estimated probabilities for the CN, MCI, and AD classes, respectively. Here c∈{CN,MCI,AD} denotes the diagnostic class index and $p_{c}=P{\left(s\right)}_{c}$ denotes the estimated probability that slice *s* belongs to class *c*. The predicted slice-level label (${\hat{y}}_{s}$) corresponds to the class with the highest estimated probability and is determined by:

(1) $${\hat{y}}_{s}=\arg\max_{c\in \{\mathrm{CN},\mathrm{MCI},\mathrm{AD}\}}p_{c}$$

This strategy assesses the model’s ability to identify diagnostically relevant features from individual anatomical cross-sections. We evaluated each of the 6353 test slices independently to characterize pattern recognition performance on isolated 2D sections.

In scan-level classification, we aggregated predictions across all slices constituting each complete MRI volume to generate a single diagnostic prediction per scan, reflecting clinical practice where radiologists review entire scan series. For a scan $S=\{s_{1},s_{2},\ldots ,s_{n}\}$, where $s_{i}$ denotes the *i*-th axial slice and *n* denotes the total number of slices, the aggregation strategy first computed class probabilities $P{\left(s_{i}\right)}_{c}$ for each constituent slice $s_{i}\in S$ and each class c∈{CN,MCI,AD} (2). The final scan-level prediction was determined by first identifying the slice $s^{*}\in S$ with the highest confidence prediction across classes:

(2) $$s^{*}=\arg\max_{s_{i}\in S}\left(\max_{c}P{\left(s_{i}\right)}_{c}\right)$$

where $P{\left(s_{i}\right)}_{c}$ denotes the estimated probability that slice $s_{i}$ belongs to class *c*. The predicted scan-level label ${\hat{y}}_{S}$ was then defined as the class *c* achieving the highest predicted probability on slice $s^{*}$:

(3) $${\hat{y}}_{S}=\arg\max_{c}P{\left(s^{*}\right)}_{c}$$

where $P{\left(s^{*}\right)}_{c}$ denotes the predicted probability that slice $s^{*}$ belongs to class *c*. The slice achieving the highest maximum class probability determined the final scan-level classification. This aggregation approach leverages information from multiple anatomical sections, potentially improving diagnostic robustness compared to single-slice decisions by reducing the impact of individual slice artifacts. We evaluated scan-level performance across 627 complete test volumes.

We generated confusion matrices for both evaluation approaches to facilitate detailed error analysis across diagnostic categories. These matrices display predicted versus true class distributions, enabling identification of systematic misclassification patterns. Diagonal elements represent correct classifications; off-diagonal elements indicate misclassifications. We paid particular attention to false negative rates, as minimizing them for AD and especially MCI represents a critical priority—delayed diagnosis compromises opportunities for early therapeutic intervention and preventive strategies that could slow disease progression.

## 3. Results

Our results are presented in this section, including an overview of model performance, architectural comparisons, detailed analysis of top models, multi-slice aggregation impact, confusion matrix analysis and comparison with the literature.

### 3.1. Overview of Model Performance

We report results for slice-level classification on 6353 test images and scan-level classification on 627 test scans. Table 4 presents complete test set results for all architectures across both evaluation approaches, revealing considerable performance heterogeneity across architectural families. Notably, certain CNN-based models outperformed Transformer architectures in classification accuracy despite the latter’s increased structural complexity.

These results demonstrate several critical findings. First, EfficientNetV2-L achieved the highest slice-level accuracy (86.84%), surpassing the baseline custom CNN by 10.15 percentage points. Second, at scan-level, ConvNeXtV2-L attained the highest accuracy (91.07%), confirming that aggregating predictions across anatomical sections enhances diagnostic reliability. Third, VGG19 demonstrated the most reliable probabilistic calibration with optimal balance between accuracy and loss at both evaluation levels, achieving the lowest loss values (0.4317 slice-level, 0.5866 scan-level) while maintaining competitive accuracy (86.07% and 88.52%, respectively).

### 3.2. Architectural Comparison

To elucidate architectural design’s impact on diagnostic performance, we grouped models into categories for comparative analysis. Table 5 presents aggregate statistics for traditional CNNs, modern and hybrid CNNs, and Transformer architectures.

This categorical analysis reveals several notable patterns. Traditional CNNs achieved the highest mean accuracy despite having the smallest average parameter counts and shortest training times, demonstrating superior computational efficiency. Modern CNNs showed comparable accuracy with moderate parameter counts but longer training times due to increased architectural complexity. Transformer-based architectures demonstrated the poorest performance despite having the largest parameter counts and longest training times, with mean accuracy approximately 6% lower than CNNs and mean loss more than double that of traditional CNNs.

These performance patterns contradict the prevalent assumption that Transformer architectures consistently outperform CNNs in computer vision tasks. Several factors may explain this substantial performance gap. First, CNNs incorporate inductive biases (local receptive fields and translation equivariance) that naturally align with hippocampal atrophy patterns appearing at consistent spatial locations following MNI normalization. Transformers lack these built-in assumptions and must learn all spatial relationships from data through attention mechanisms. While this flexibility enables pattern discovery in massive natural image datasets, our medical imaging dataset (20,676 training images compared to ImageNet’s 1.2 million) may be insufficient for attention mechanisms to discover effective spatial strategies that CNNs obtain through architectural design. These results indicate that medical imaging applications involving structured anatomical patterns benefit substantially from convolutional networks’ inherent inductive biases. Second, diagnostic features in our task are predominantly local (hippocampal volumetric reduction, ventricular enlargement, tissue boundary alterations), which CNNs naturally capture through their hierarchical local feature extraction. Transformers’ global attention mechanisms excel at modeling long-range dependencies but may provide limited advantage when diagnostic information resides primarily in localized anatomical structures emphasized by our hippocampal slice extraction strategy.

Furthermore, DINOv2-L’s weak performance despite self-supervised pre-training on 142 million images reveals limitations in transferring knowledge from natural to medical images. DINOv2’s particularly poor results demonstrate that even massive-scale pre-training cannot overcome domain mismatch when natural RGB images (object textures, color distributions) diverge fundamentally from grayscale medical scans (tissue intensity patterns, anatomical boundaries). Large-scale pre-training on natural RGB imagery appears insufficient to address the distinct visual properties of grayscale medical scans, regardless of dataset size. DINOv2-L’s limitations extend beyond data type differences. Its self-supervised training objective (learning invariance to data augmentations) may reduce sensitivity to subtle intensity differences and texture patterns critical for medical imaging. Self-supervised training may inadvertently treat these diagnostically relevant subtle features as noise. Moreover, even when fine-tuned, the 304.89 M parameters originally optimized for natural images adapt poorly to medical imaging. This suggests that self-supervised pre-training objectives may not align well with medical imaging task requirements.

### 3.3. Detailed Analysis of Top-Performing Models

Table 6 presents per-class metrics for the three optimal architectures at slice-level.

Per-class analysis reveals clinically significant performance patterns. EfficientNetV2-L demonstrated the most balanced performance across diagnostic categories, with precision and recall ranging from 0.84 to 0.89, indicating reliable classification regardless of disease stage. Notably, EfficientNetV2-L achieved the highest MCI recall (0.88), correctly identifying 2006 of 2275 MCI slices with only 269 false negatives. Detecting prodromal disease stages (MCI) represents the highest clinical priority, as this stage offers the optimal window for interventions that may delay progression to Alzheimer’s dementia.

VGG19 demonstrated impressive AD detection performance with the highest recall (0.91), correctly classifying 1771 of 1944 AD slices with only 173 false negatives. This is clinically crucial, as missed AD diagnoses delay essential care interventions and patient support. However, VGG19 showed lower CN recall (0.81) with 409 misclassifications.

ConvNeXtV2-L showed more uniform but slightly lower performance across classes, with recall ranging from 0.83 to 0.87. This model produced the highest total false negatives (976) across all classes. While ConvNeXtV2-L achieved strong overall accuracy, its per-class reliability fell slightly below the other top performers.

Table 7 presents scan-level metrics on 627 test scans, revealing improved performance through aggregation.

Scan-level aggregation produced substantial performance improvements across all models and classes. ConvNeXtV2-L achieved the highest overall accuracy (91.07%), representing a 6.43% improvement over its slice-level performance. Furthermore, ConvNeXtV2-L achieved AD recall of 0.96, correctly identifying 104 of 108 AD scans with only 4 false negatives. This performance approaches clinically acceptable thresholds for diagnostic support systems.

VGG19 demonstrated CN recall of 0.93 at scan-level, correctly classifying 181 of 194 cognitively normal scans with only 13 false negatives. VGG19 also showed balanced overall performance with macro-averaged recall of 0.89 and the second-lowest total false negatives (72).

EfficientNetV2-L achieved the highest scan-level MCI recall (0.93), correctly identifying 301 of 325 MCI scans with only 24 false negatives. However, this model showed weaker AD and CN detection with recall of 0.76 for both classes, producing 26 false negative AD cases and 47 false negative CN cases. EfficientNetV2-L unexpectedly showed degraded scan-level performance compared to slice-level results, suggesting the aggregation strategy may not effectively leverage this architecture’s learned features.

### 3.4. Impact of Multi-Slice Aggregation

The performance enhancements observed with scan-level aggregation warrant in-depth analysis. Table 8 compares slice-level and scan-level performance for the five models evaluated under both strategies.

Aggregation proved most beneficial for ConvNeXtV2-L and SwinV1-L, with accuracy gains of 7.60% and 9.04%, respectively. These improvements suggest both models produce variable prediction confidence across anatomical locations. Certain slices containing more pronounced pathological features yield high-confidence predictions for scan-level diagnosis. For SwinV1-L, the Transformer architecture appears especially well suited to this strategy, generating diverse prediction confidence across scan volumes, where some slices produce strong classifications while others exhibit lower confidence. By selecting the maximum-confidence prediction, the aggregation method effectively exploits this variability, filtering uncertain decisions and retaining the most reliable prediction.

In contrast, modest aggregation gains for VGG architectures (1.45% to 2.85%) may reflect more consistent predictions across slices with minimal confidence variation between anatomical locations. This consistency limits maximum-confidence aggregation effectiveness, as distinguishing between slices with similarly high confidence becomes difficult. These results reflect VGG architecture’s inherent characteristics—convolutional layers process all spatial locations uniformly, lacking the regional focus that attention mechanisms provide.

**Figure 7:**
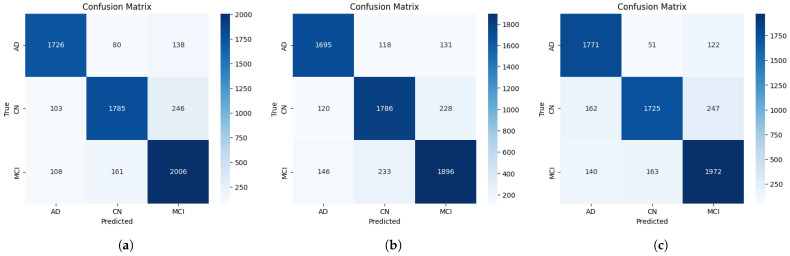
Confusion matrices for EfficientNetV2-L (**a**), ConvNeXtV2-L (**b**) and VGG19 (**c**) at slice-level. The diagonal with darker cells represents the correct predictions and the lighter cells indicates the misclassifications.

In contrast, EfficientNetV2-L demonstrated a 2.66% performance decline at scan-level, concerning behavior warranting closer inspection. Despite achieving the highest slice-level accuracy (86.84%), scan-level degradation to 84.53% indicates problematic volumetric generalization. This pattern likely reflects poorly calibrated confidence: EfficientNetV2-L occasionally generates highly confident but incorrect predictions on specific slices, which maximum-confidence aggregation systematically selects for scan-level classification. When confidence estimates are unreliable, this strategy may amplify rather than mitigate errors. This is evident in Figure 7, where scan-level EfficientNetV2-L shows elevated MCI errors (20 AD-to-MCI, 40 CN-to-MCI), suggesting the model generates overconfident MCI predictions that dominate aggregation even when incorrect. This contrasts with ConvNeXtV2-L and SwinV1-L, whose confidence estimates align more reliably with prediction correctness, enabling aggregation to select genuinely informative slices. Maximum-confidence aggregation effectiveness therefore depends critically on model calibration—it benefits models with reliable confidence estimates but degrades accuracy when confidence is poorly calibrated by systematically selecting overconfident errors.

Differential aggregation responses across architectures reveal fundamental differences in how models distribute confidence across anatomical sections. Transformers and hybrid architectures (ConvNeXtV2-L, SwinV1-L) employ attention mechanisms creating heterogeneous spatial processing—assigning high attention weights to slices with salient pathological features while processing others with lower confidence. This prediction strength variability makes maximum-confidence aggregation effective: selecting slices where the model exhibits strongest activation identifies anatomical sections with the most discriminative features, yielding substantial gains (+7.60% and +9.04%, respectively).

In contrast, traditional CNN architectures (VGG16, VGG19) process all spatial locations uniformly through fixed convolutional hierarchies without attention modulation, producing relatively consistent predictions across adjacent slices. When most slices generate similar confidence scores, maximum-confidence selection provides limited advantage over averaging, explaining modest gains (+1.45% to +2.85%).

EfficientNetV2-L represents a distinct failure mode: its compound scaling and squeeze-and-excitation mechanisms produce confidence variability across slices, but this variability does not correlate with prediction correctness. The aggregation strategy therefore systematically selects incorrect high-confidence predictions, degrading performance (−2.66%).

Multi-slice maximum-confidence aggregation effectiveness depends on model architecture. It benefits models with reliable confidence estimates (SwinV1-L, ConvNeXtV2-L) but degrades accuracy in overconfidence-prone models like EfficientNetV2-L by systematically selecting overconfident errors. Clinical deployment therefore requires either careful model selection with verified confidence reliability or adoption of more robust aggregation methods (majority voting, weighted averaging, ensemble approaches).

### 3.5. Performance Evaluation and Error Analysis Using Confusion Matrices

**Figure 8:**
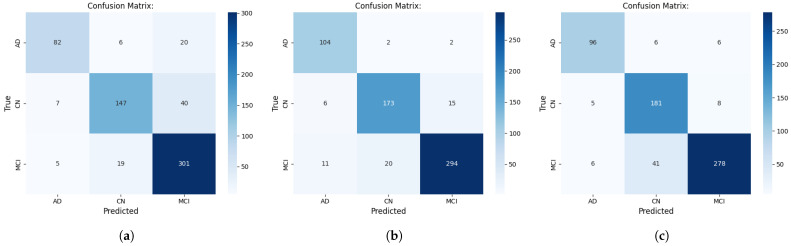
Confusion matrices for EfficientNetV2-L (**a**), ConvNeXtV2-L (**b**) and VGG19 (**c**) at scan-level. The diagonal with darker cells represents the correct predictions and the lighter cells indicates the misclassifications.

Confusion matrices for the three top-performing models enable identification of classification behaviors. Figure 7 and Figure 8 present slice-level and scan-level confusion matrices, respectively.

#### 3.5.1. Slice-Level Confusion Matrix Analysis

Across all three models, MCI classification achieved the highest per-class accuracy: EfficientNetV2-L correctly identified 2006 of 2275 MCI slices (88.17%), VGG19 classified 1972 (86.68%), and ConvNeXtV2-L identified 1896 (83.34%). Although MCI lies between normal cognition and AD, learned representations remained clearly distinguishable. These results indicate learned representations enabled MCI differentiation from both CN and AD at slice-level. However, this does not imply direct neuroanatomical biomarker identification, as we performed no region-level attribution analysis.

The primary error source across all models involved bidirectional MCI-CN confusion, mirroring the clinical difficulty in distinguishing these groups. VGG19 incorrectly labeled 247 CN slices as MCI (11.57% of 2134 CN cases) and 163 MCI slices as CN (7.16% of 2275 MCI cases). EfficientNetV2-L displayed comparable patterns: 246 CN-to-MCI (11.53%) and 161 MCI-to-CN (7.08%). ConvNeXtV2-L showed the highest MCI-to-CN confusion (233 cases, 10.24%). This bidirectional error pattern between adjacent diagnostic categories represented the most frequent classification challenge across all architectures.

AD misclassifications were less frequent and aligned with disease progression, occurring primarily between AD and MCI rather than CN. VGG19 generated 122 AD-to-MCI errors (6.27%) versus only 51 AD-to-CN errors (2.62%). EfficientNetV2-L showed 138 AD-to-MCI (7.1%) versus 80 AD-to-CN (4.12%). ConvNeXtV2-L produced 131 (6.74%) versus 118 (6.07%), respectively. Lower AD-to-CN confusion compared to AD-to-MCI confusion is consistent with expected diagnostic category continuity. However, we performed no direct anatomical validation to confirm the structural basis of these distinctions. VGG19’s low AD-to-CN rate (2.62%) may demonstrate stable misdiagnosis avoidance, although higher CN-to-AD confusion (162 cases, 7.59%) may indicate overclassification of normal scans as pathological.

#### 3.5.2. Scan-Level Confusion Matrix Analysis

Scan-level aggregation generally reduced misclassification rates, though the magnitude varied by model and diagnostic class. ConvNeXtV2-L achieved notable performance: 104 of 108 AD scans (96.3%), 173 of 194 CN scans (89.18%), and 294 of 325 MCI scans (90.46%) correctly identified. This AD performance is reflected in only 2 AD-to-CN (1.85%) and 2 AD-to-MCI (1.85%) confusions, representing substantial reductions from slice-level errors. The remaining AD misclassifications reflect residual overlap between diagnostic categories at scan-level.

At scan-level, VGG19 showed improved CN classification accuracy, correctly identifying 181 of 194 CN scans (93.3%). CN misclassifications decreased significantly: CN-to-MCI dropped to 8 cases (4.12%) and CN-to-AD to 5 cases (2.57%). These improvements suggest VGG19 may be especially suitable for screening programs where accurately identifying healthy individuals is the primary objective. However, MCI-to-CN confusion remained elevated (41 cases, 12.62%), indicating persistent difficulty with prodromal cases despite multi-slice aggregation. AD errors were balanced: 6 AD-to-CN and 6 AD-to-MCI (5.6% each).

EfficientNetV2-L showed unexpected scan-level performance characterized by error redistribution. MCI performance improved, with 301 of 325 scans correctly identified (92.62%)—the best among all models. However, AD and CN accuracies declined. AD produced 20 AD-to-MCI errors (18.52%), though AD-to-CN errors dropped to 6 (5.6%). CN classification was more affected: 40 CN-to-MCI errors (20.62%) and 7 CN-to-AD errors (3.61%). These results indicate EfficientNetV2-L’s systematic tendency to overpredict MCI at scan-level under the current aggregation strategy. This behavior likely reflects model-specific confidence distribution characteristics rather than disease-related patterns.

Confusion matrices demonstrate that disease stage separability varies across architectures. All three models distinguished AD and CN at scan-level with minimal confusion. However, MCI classification remained the most variable. ConvNeXtV2-L achieved relatively balanced MCI confusion (11 toward AD, 20 toward CN). VGG19 demonstrated asymmetric error distribution (6 toward AD, 41 toward CN). EfficientNetV2-L overpredicted MCI (20 and 40 cases for AD and CN, respectively).

Overall, confusion matrices demonstrate that class separability differs across architectures, particularly for MCI. ConvNeXtV2-L shows relatively balanced error distribution across adjacent classes, whereas VGG19 and EfficientNetV2-L exhibit asymmetric misclassification tendencies. These findings highlight the importance of evaluating not only overall accuracy but also class-wise error structure when comparing architectures.

### 3.6. Related Work and Performance Comparison

Table 9 summarizes selected studies performing three-class CN/MCI/AD classification using ADNI MRI data to contextualize our findings. However, direct numerical comparison across studies requires substantial caution due to marked methodological heterogeneity. Methodological differences include dimensionality (2D slice-based vs. 3D volumetric models), evaluation level (slice-level vs. patient-level), temporal inputs (cross-sectional vs. longitudinal), preprocessing pipelines, segmentation strategies, architectural complexity, ensemble integration, and data splitting methodology. These factors significantly influence reported accuracy, limiting the validity of ranking studies solely by percentage performance.

Overall, our slice-level accuracies (84–87%) fall within the range reported for 2D CNN-based MRI studies such as Lim et al. (78.57%) [8], Chen et al. (84.37%) [31], Basheera (86.70%) [32], and Savaş et al. (80.19–90.88%) [34]. However, these studies differ in slice selection strategies, segmentation procedures, normalization techniques, and validation protocols—factors that substantially affect performance. Consequently, similar reported accuracies do not imply methodological equivalence.

At the patient level, ConvNeXtV2-L achieved 91.07% accuracy. This lies within the range reported by patient-level studies such as Sahumbaiev et al. (88.31%) [33], El-Sappagh et al. (93.87%) [35], and Alindo et al. (94.25%) [7]. Nevertheless, several higher-performing approaches incorporate methodological elements absent from our framework, including 3D volumetric convolutional networks [33], multimodal or longitudinal modeling with temporal architectures [35], segmentation-enhanced pipelines [32], or ensemble-based classifiers trained on multiple representations [36]. These methodological differences preclude direct performance equivalence.

Evaluation protocol discrepancies represent a major variation source across the literature. Our study strictly enforced scan-level data separation, ensuring all slices from a given scan belonged exclusively to either training or test sets. In contrast, slice-level random splitting may inadvertently introduce information leakage due to high anatomical similarity between adjacent slices from the same individual, potentially inflating accuracy estimates. Moreover, preprocessing pipelines vary considerably across studies, including ICA segmentation [32], 3D-to-2D projection strategies [7], and cross-dataset ensemble validation [36], each introducing distinct inductive biases.

For these reasons, Table 9’s comparison should be interpreted contextually rather than hierarchically. The objective is not to claim superiority but to situate our anatomically targeted 2D single-plane framework within the spectrum of methodological approaches in the ADNI MRI literature.

Our findings demonstrate that a localized medial temporal slice-based strategy with scan-level separation achieves performance within the distribution of cross-sectional MRI classification studies. However, definitive positioning relative to state-of-the-art requires harmonized benchmarking protocols, standardized data splits, and controlled evaluation settings across studies. Future research should prioritize reproducible multi-study benchmarks for more rigorous methodological comparison.

## 4. Discussion

We interpret findings in clinical context and examine study limitations below.

From a health promotion perspective, early MCI detection represents the greatest opportunity for intervention in Alzheimer’s disease progression. EfficientNetV2-L achieved 92.6% MCI recall at scan-level, detecting 301 of 325 early-stage patients with only 24 missed cases. Each missed diagnosis constitutes a lost opportunity for therapeutic intervention when treatments are most effective at slowing disease progression. Population health implications are substantial. With dementia cases projected to reach 139 million by 2050, even small improvements can have substantial impact on daily life. A 10% increase in AI-assisted MCI detection combined with 10% reduction in progression could potentially prevent hundreds of Alzheimer’s cases. Computational efficiency becomes crucial for global healthcare deployment, requiring balance between performance and real-world application constraints. Models like VGG19, with low resource requirements, could be well suited for clinical deployment. Enabling AI-assisted early detection may reduce global healthcare disparities by offering access to these technologies, particularly with portable low-cost MRI for screening programs.

Medical image classification remains challenging, requiring medical expertise for proper interpretation. Results indicate that larger, more modern architectures do not always yield better performance, as evidenced by DINOv2-L (72.47% despite 304.89 M parameters). Model size and parameter count alone are insufficient performance indicators. Input image size helped some models but not others, depending on their backbones. While EfficientNetV2-L benefited from 384×384 resolution, other architectures showed minimal gains from larger inputs. What matters most is balance between efficient architecture, appropriate complexity level, and suitable pretraining. Pre-training proved to be an important factor. Models pretrained on large datasets like ImageNet [37] (e.g., VGG) achieved lower losses and more stable training compared to models trained from scratch or with inadequate pre-training (e.g., DINOv2-L using self-supervised training). CNN-Transformer performance comparisons illustrate practical trade-offs. Traditional CNN architectures, despite their simplicity, showed strong results and often outperformed larger models while being faster and computationally less expensive. In contrast, Transformers converged quickly in terms of epochs but required substantially more computational resources, challenging their integration into real-world healthcare applications. Ensuring model robustness proved challenging, as some techniques showed limited effectiveness. Data augmentation techniques (random flips, rotations, color jitter) aimed to improve generalization by increasing effective training set diversity. However, preliminary experiments revealed minimal impact on validation accuracy compared to models without augmentation, suggesting our rigorous preprocessing pipeline already provided sufficient standardization for robust feature learning.

A significant limitation is scan-level rather than subject-level data partitioning. The ADNI cohort contains multiple longitudinal scans per subject, and our splitting approach allowed different scans from the same individual in both training and test sets, introducing potential temporal overlap. However, several factors mitigate this concern:

- Our MNI normalization pipeline explicitly removes subject-specific anatomical signatures, forcing models to learn disease-related features rather than patient characteristics.
- Our scan-level accuracy (91.07%) falls within the range of comparable ADNI studies (Table 9), not the near-perfect performance expected from patient memorization.
- Confusion matrices show biologically plausible errors concentrated between adjacent disease stages, consistent with clinical difficulty rather than memorization artifacts.
- Most critically, our comparative findings (traditional CNNs outperform Transformers; preprocessing quality impacts reliability; scan-level aggregation improves accuracy) remain valid because all fifteen architectures were evaluated under identical conditions.

Notably, this limitation appears underreported in ADNI-based research—review of comparable studies reveals many do not explicitly clarify whether splits enforce subject-level independence. Our transparent acknowledgment contributes to improving methodological reporting standards. Future ADNI studies should explicitly document split strategies, longitudinal visit handling, and temporal overlap. While this limitation affects absolute performance estimates, it does not invalidate our relative architectural insights, which constitute this work’s primary scientific contribution. Future studies should prioritize subject-level partitioning.

Variable slice extraction (CN: 11, MCI: 7, AD: 18 slices per scan) balanced the training dataset at image level, compensating for scan-level imbalance. This ensured equal class representation, preventing learning biases. We acknowledge variable slice counts could theoretically influence scan-level predictions by providing different numbers of opportunities for confident classifications. However, confusion patterns show biologically plausible errors between adjacent disease stages rather than systematic artifacts. Future work could explore fixed extraction with weighted sampling or ablation studies quantifying this choice’s impact.

The selected axial plane captures key AD structural biomarkers. Symmetric regional sampling around this coordinate reduces sensitivity to minor registration variability. While a single-plane approach cannot represent neurodegeneration’s full three-dimensional extent, our results indicate localized medial temporal information provides sufficient discriminative signal for three-way classification. Future work will evaluate slice-position robustness and multi-plane or lightweight 3D strategies to enhance generalizability.

At scan-level, we derived predictions using maximum-evidence aggregation, where the slice with highest class confidence determines the final label. This reflects the assumption that focal structural alterations in Alzheimer’s disease may be most discriminative at specific levels. While this approach emphasizes the strongest localized signal within a scan, its effectiveness depends on slice-level confidence calibration. Future work should compare aggregation strategies (probability averaging, hierarchical slice-to-scan models) to improve robustness.

Age, sex, and scanner manufacturer were not explicitly controlled in stratified evaluations. Age distributions are balanced across groups. Standardized ADNI acquisition protocols combined with our preprocessing pipeline reduce scanner-related variability. Nevertheless, residual confounding cannot be excluded. Future work should include stratified analyses and covariate regression to quantify these factors’ impact on model predictions.

An additional limitation is the absence of ablation studies quantifying each preprocessing step’s contribution to performance. While our pipeline incorporates technical parameters validated in prior neuroimaging work, we did not empirically validate whether these specific choices or alternative implementations would yield equivalent classification results. Future work should conduct ablation experiments isolating individual preprocessing components to establish their performance impact.

Our evaluation employs per-class precision, recall, F1-scores, and confusion matrices but excludes ROC or Precision-Recall curves. For three-way classification, such curves require decomposition into multiple binary problems, generating numerous visualizations that would complicate comparative analysis across fifteen architectures. Our current metrics provide equivalent diagnostic information at standard deployment thresholds and align with evaluation approaches in comparable literature.

Another important limitation is the absence of external validation on independent datasets beyond ADNI. While our controlled ADNI comparison isolates architectural effects from preprocessing confounds, generalization to different acquisition protocols, scanner manufacturers, or population demographics remains unvalidated. Future work should evaluate our findings on external datasets (OASIS, AIBL) to assess whether CNNs’ superiority over Transformers and architecture-dependent aggregation patterns persist across data distributions.

## 5. Conclusions

The public health imperative is clear: 10–15% annual MCI-to-AD conversion and projected 139 million dementia cases by 2050. Improving early MCI detection could enable thousands to receive treatments when most effective, potentially preventing progression to Alzheimer’s disease. This work provides systematic comparative evaluation of fifteen deep learning architectures for classifying cognitively normal (CN), mild cognitive impairment (MCI), and Alzheimer’s disease (AD) subjects using the ADNI dataset. Our methodological contribution introduces a dual-level evaluation framework revealing architecture-dependent aggregation effects under standardized conditions.

Our dual-level framework demonstrates asymmetric multi-slice aggregation effects across architectures. ConvNeXtV2-L and SwinV1-L exhibited scan-level improvements of 7.60% and 9.04%, respectively, while EfficientNetV2-L experienced 2.66% degradation. This establishes architectural selection and aggregation strategy as interdependent factors for clinical deployment. CNNs and hybrid pre-trained models outperformed Transformer-based models at both slice and scan levels. EfficientNetV2-L achieved the highest slice-level accuracy (86.84%), ConvNeXtV2-L the best scan-level performance (91.07%), and VGG19 optimal balance (86.07% slice/88.52% scan). Traditional CNNs achieved higher performance than Vision Transformers despite larger parameter counts and longer training times, indicating architecture characteristics matter more than model size for medical imaging tasks with localized features.

Model performance depends strongly on data selection and preprocessing. Focusing on hippocampal axial slices proved efficient but may miss changes in other sections. Future work should explore the incorporation of coronal and sagittal views, full 3D scans, or cropped regions of interest to improve accuracy while maintaining performance.

Due to scan-level rather than subject-level data partitioning, the reported accuracy values should not be interpreted as fully independent diagnostic benchmarks. While this design may influence absolute performance estimation, the controlled comparative evaluation under identical preprocessing and training conditions preserves the internal consistency of the architectural analysis. Future work should enforce strict subject-level partitioning to provide unbiased performance validation.

This study confirms CNNs as effective frameworks for hippocampal MRI analysis. Integrating multiple imaging modalities (e.g., PET) may enhance diagnostic quality and enable AI-assisted clinical integration. However, the proposed techniques with low resource requirements may facilitate screening programs with portable, low-cost MRI.

## Figures and Tables

**Table 1:** Demographic summary of the studied cohort.

	CN	MCI	AD
Number of Subjects	195	311	133
Female/Male	93/102	110/201	64/69
Number of Scans	984	1575	575
Age (mean ± SD)	77.07 ± 5.07	75.92 ± 7.06	75.63 ± 7.61

**Table 2:** Summary of the number of images comprising the dataset used in this study.

	CN	MCI	AD	Total
Number of Subjects	195	311	133	639
Number of Scans	984	1575	575	3134
Number of Slices	10,824	11,025	10,350	32,199

**Table 3:** Comparative performance of deep learning architectures for brain MRI classification. Architectural characteristics define the methodological scope of our evaluation framework. Test performance metrics (detailed in Section 3) facilitate comparison of complexity-performance trade-offs. Epoch counts varied across models due to early stopping based on validation loss convergence, ensuring each architecture reached optimal performance rather than imposing arbitrary uniform training duration. Training times were measured on Google Colab Pro (NVIDIA A100 GPU, 40 GB memory, 83.5 GB RAM).

Model	Test Acc.	Test Loss	Parameters	Training Time	Epochs	Pre-Training	Input Size
CNN (Scratch)	0.7669	0.7234	4.37 M	5 h 06 min	520	–	256 × 256
VGG16	**0.8568**	0.4501	14.88 M	1 h 31 min	150	ImageNet	224 × 224
VGG19	**0.8607**	0.4317	20.19 M	2 h 18 min	160	ImageNet	224 × 224
MobileNetV2	0.6931	2.4713	2.59 M	2 h 12 min	185	ImageNet	224 × 224
InceptionV3	0.7914	0.7416	22.36 M	1 h 51 min	185	ImageNet	299 × 299
EfficientNetB0	0.7776	1.1750	4.38 M	2 h 01 min	190	ImageNet	224 × 224
EfficientNetB2	0.8146	1.1117	8.13 M	2 h 32 min	200	ImageNet	260 × 260
EfficientNetV2B2	0.8180	0.9788	9.13 M	2 h 40 min	200	ImageNet	224 × 224
EfficientNetV2-L	**0.8684**	0.7132	118.4 M	8 h 08 min	200	ImageNet-21k	384 × 384
DenseNet201	0.7241	0.9291	18.85 M	2 h 15 min	170	ImageNet	224 × 224
ConvNeXtV2-L	**0.8464**	1.1980	196.42 M	9 h 14 min	65	ImageNet-22k	224 × 224
ViT-Base/16	0.6591	2.6725	85.8 M	3 h 10 min	65	ImageNet-21k	224 × 224
SwinV1-L	**0.8264**	1.0586	195 M	8 h 47 min	60	ImageNet-22k	224 × 224
SwinV2-L	0.8048	1.5402	195.2 M	9 h 50 min	60	ImageNet-22k	224 × 224
DinoV2-L	0.7247	3.1354	304.89 M	11 h 27 min	60	LVD-142 M	224 × 224

Boldface values indicate particularly important or noteworthy results.

**Table 4:** Complete test set performance results across all evaluated architectures.

Model	Architecture Type	Slice Acc.	Slice Loss	Scan Acc.	Scan Loss
CNN (scratch)	Custom CNN	0.7669	0.7234	–	–
VGG16	Traditional CNN	**0.8568**	**0.4501**	0.8692	**0.7165**
VGG19	Traditional CNN	**0.8607**	**0.4317**	**0.8852**	**0.5866**
MobileNetV2	Efficient CNN	0.6931	2.4713	–	–
InceptionV3	Multi-scale CNN	0.7914	0.7416	–	–
EfficientNetB0	Compound-scaled CNN	0.7776	1.1750	–	–
EfficientNetB2	Compound-scaled CNN	0.8146	1.1117	–	–
EfficientNetV2B2	Efficient CNN v2	0.8180	0.9788	–	–
EfficientNetV2-L	Efficient CNN v2	**0.8684**	**0.7132**	0.8453	**0.9141**
DenseNet201	Dense CNN	0.7241	0.9291	–	–
ConvNeXtV2-L	Hybrid CNN-Transformer	0.8464	1.1980	**0.9107**	1.2320
ViT-Base/16	Pure Transformer	0.6591	2.6725	–	–
SwinV1-L	Hierarchical Transformer	0.8264	1.0586	**0.9011**	1.0117
SwinV2-L	Hierarchical Transformer v2	0.8048	1.5402	–	–
DINOv2-L	Self-supervised Transformer	0.7247	3.1354	–	–

Scan-level evaluation was conducted only for the five highest-performing models in slice-level classification. Bold values indicate top three performances in each category.

**Table 5:** Performance comparison across architectural categories.

Architecture Category	Models	Mean Slice Acc.	Mean Slice Loss	Mean Parameters	Mean Training Time ^1^
Traditional CNNs	VGG16, VGG19, DenseNet201	0.8139	0.6036	17.97 M	2 h 01 min
Modern & Hybrids CNNs	MobileNetV2, InceptionV3, EfficientNet family, ConvNeXtV2-L	0.8014	1.1985	51.63 M	4 h 05 min
Transformers	ViT-Base/16, Swin family, DINOv2-L	0.7538	2.1017	195.22 M	8 h 18 min

^1^ Training times measured on Google Colab Pro (NVIDIA A100 GPU with 40 GB memory, 83.5 GB RAM).

**Table 6:** Detailed per-class performance metrics for top-performing models at slice-level.

Model	Class	Accuracy	Precision	Recall	F1-Score	Support	False Neg.
EfficientNetV2-L	AD	0.87	0.89	0.89	0.89	1944	218
	CN	0.87	0.88	0.84	0.86	2134	349
	MCI	0.87	0.84	0.88	0.86	2275	269
	Macro Avg	0.87	0.87	0.87	0.87	6353	836
VGG19	AD	0.86	0.85	0.91	0.88	1944	173
	CN	0.86	0.89	0.81	0.85	2134	409
	MCI	0.86	0.84	0.87	0.85	2275	303
	Macro Avg	0.86	0.86	0.86	0.86	6353	885
ConvNeXtV2-L	AD	0.85	0.86	0.87	0.87	1944	249
	CN	0.85	0.84	0.84	0.84	2134	348
	MCI	0.85	0.84	0.83	0.84	2275	379
	Macro Avg	0.85	0.85	0.85	0.84	6353	976

Per-class performance metrics for the three best models evaluated on 6353 test slices. False negatives represent missed diagnoses, which are particularly critical for early detection of AD and MCI. Macro averages provide overall model performance across all classes.

**Table 7:** Detailed per-class performance metrics for top-performing models at scan-level.

Model	Class	Accuracy	Precision	Recall	F1-Score	False Neg.
EfficientNetV2-L	AD	0.85	0.87	0.76	0.81	26
	CN	0.85	0.85	0.76	0.80	47
	MCI	0.85	0.83	0.93	0.88	24
	Macro Avg	0.85	0.85	0.82	0.83	97
VGG19	AD	0.89	0.90	0.89	0.89	12
	CN	0.89	0.79	0.93	0.86	13
	MCI	0.89	0.95	0.86	0.90	47
	Macro Avg	0.89	0.88	0.89	0.88	72
ConvNeXtV2-L	AD	0.91	0.86	0.96	0.91	4
	CN	0.91	0.89	0.89	0.89	21
	MCI	0.91	0.95	0.90	0.92	31
	Macro Avg	0.91	0.90	0.92	0.91	56

Per-class performance metrics for the three best models evaluated at scan-level across 627 test volumes. ConvNeXtV2-L achieved the highest accuracy (91.07%) with the lowest total false negatives (56).

**Table 8:** Comparison of slice-level and scan-level accuracy with relative performance gains.

Model	Slice Accuracy	Scan Accuracy	Accuracy Delta (%)
VGG16	0.8568	0.8692	+1.45%
VGG19	0.8607	0.8852	+2.85%
EfficientNetV2-L	0.8684	0.8453	−2.66%
SwinV1-L	0.8264	**0.9011**	**+9.04%**
ConvNeXtV2-L	0.8464	**0.9107**	**+7.60%**

Accuracy delta indicates percentage improvement (positive values) or decline (negative values) from slice-level to scan-level evaluation. Boldface values indicate particularly important or noteworthy results.

**Table 9:** Comparative Analysis with State-of-the-Art Methods on CN vs. MCI vs. AD Classification Using MRI Images. (S) indicates slice-level accuracy and (P) indicates patient-level accuracy.

Study	Year	Approach	Accuracy (%)	Methodology
Lim et al. [8]	2022	VGG-16	78.57 (I)	Similar preprocessing pipeline
Chen et al. [31]	2024	Ensemble (Faster R-CNN, Soft-NMS, ResNet50)	84.37 (P)	Ensemble with Soft-NMS integration
Basheera [32]	2019	CNN (segmented gray matter + ICA)	86.70 (I)	Enhanced ICA segmentation
Wong et al. [28]	2023	EfficientNetB0 (Transfer Learning)	87.17 (I)	Transfer learning focus
Sahumbaiev et al. [33]	2018	HadNet	88.31 (P)	3D volumetric CNN (HadNet)
Savaş et al. [34]	2022	MobileNetV2	80.19 (I)	Multiple architecture comparison
Savaş et al. [34]	2022	VGG19	89.77 (I)	Multiple architecture comparison
Savaş et al. [34]	2022	EfficientNetB2	90.88 (I)	Multiple architecture comparison
Alindo et al. [7]	2023	VGG19	90.94 (I)	3D-to-2D with segmentation and normalization
El-Sappagh et al. [35]	2022	LSTM	93.87 (P)	LSTM on longitudinal multimodal data
Alindo et al. [7]	2023	VGG16	94.25 (I)	3D-to-2D with segmentation and normalization
Loddo et al. [36]	2022	Ensemble (AlexNet, ResNet101, InceptionResNetV2)	99.22 (I)	Three-model ensemble with cross-dataset validation
Current study	2025	EfficientNetV2-L	86.84 (I)/84.53 (P) ^2^	Enhanced preprocessing and dual-level evaluation
Current study	2025	VGG19	86.07 (I)/88.52 (P) ^2^	Enhanced preprocessing and dual-level evaluation
Current study	2025	ConvNeXtV2-L	**84.64 (I)/91.07** (P) ^2^	Enhanced preprocessing and dual-level evaluation

^2^ For the current study, two accuracy values are reported corresponding to slice-level accuracy (individual 2D slices) and scan-level accuracy (aggregated predictions per patient scan), respectively. Boldface values indicate results obtained in the present work.

## Data Availability

Data used in preparation of this article were obtained from the Alzheimer’s Disease Neuroimaging Initiative (ADNI) database (adni.loni.usc.edu, accessed on 22 May 2025). As such, the investigators within the ADNI contributed to the design and implementation of ADNI and/or provided data but did not participate in the analysis or writing of this report. A complete listing of ADNI investigators can be found at: http://adni.loni.usc.edu/wpcontent/uploads/how_to_apply/ADNI_Acknowledgement_List.pdf, accessed on 21 May 2025.

## Abbreviations

The following abbreviations are used in this manuscript:

ADNI	Alzheimer’s Disease Neuroimaging Initiative
CN	Cognitively Normal
MCI	Mild Cognitive Impairment
AD	Alzheimer’s disease
MRI	Magnetic Resonance Imaging
CNN	Convolutional Neural Networks
NIfTI	Neuroimaging Informatics Technology Initiative
MPRAGE	Magnetization-Prepared Rapid Gradient-Echo
MNI	Montreal Neurological Institute
HMRF	Hidden Markov Random Field
CSF	Corticospinal Fluid
WM	White Matter
GM	Gray Matter
PET	Positron Emission Tomography
